# Supplementary material for: Strengthening the Reproductive Endocrinology and Infertility Curriculum Through Three Interactive Cases
Source: MedEdPORTAL. 2023 Dec 21;19:11375. doi: 10.15766/mep_2374-8265.11375 (PMC10733559; doi:10.15766/mep_2374-8265.11375)
Supplement: Supplementary file 1 — Learner Guide and Pre- and Postsurveys.docxFacilitator Guide.docxDelayed Postsurvey.docx [file mep_2374-8265.11375-s001.zip › B. Facilitator Guide.docx]

**Facilitator Guide – curriculum is intended to be implemented in a two-hour didactic session with OB/GYN residents and/or medical students.**

*Answers to pre- and post-survey multiple choice questions are included at the end of the document.*

***In-person***

1. Prior to session, print out a Learner Guide (A. Learner Guide.docx) for each learner, and a Facilitator Guide (B. Facilitator Guide.docx) for each facilitator.
2. Divide the learners into evenly distributed groups based on your number of facilitators. Try to achieve a range in class years, if possible.
3. Distribute the Learner Guide to learners. Allow 10 minutes for learners to independently complete pre-survey.
4. Begin with Case 1 and request a learner read the non-bolded introductory information. Then, address each bolded question to the subsequent learner, going in order around the group. Each learner will attempt to answer the question and, if unable to, the group can discuss to try to find a response together. If the question contains multiple parts, such as a question with a broad differential, the facilitator can divide it between learners, i.e., go around and have each learner name one possible diagnosis. When learners are unable to identity the answer, facilitator should help guide them to the correct answer.
5. Continue through Case 2 and Case 3 in the same manner. Offer the group a 5-minute break in between cases. Case 1 tends to be the longest and takes just over 30 minutes, case 2 takes around 30 minutes, and case 3 tends to be the shortest and takes just under 30 minutes.
6. Allow learners 10 minutes to complete the post-survey.
7. Have learners remove the surveys from the beginning and end of the Learner Guide, allowing them to keep the middle portion and any notes they took as reference. Staple the pre- and post-surveys together.
8. Email the Facilitator Guide to the learners following the curriculum to use for reference.
9. You have the option to send the delayed post-survey (DelayedPostSurvey.docx) 4-8 weeks following the completion of the curriculum to assess sustained learning.

***Virtual***

1. Prior to session, email out a Learner Guide for each learner, and a Facilitator Guide for each facilitator.
2. Divide the learners into evenly distributed groups based on your number of facilitators. Try to achieve a range in class years, if possible. You can provide separate video platform links to each group in advance. Smaller group sizes (up to 4 learners) are preferred in a virtual format, to promote engagement and participation.
3. At the beginning of the session, allow 10 minutes for learners to independently complete the pre-survey.
4. Pull up the Learner Guide and share on the screen. Have this Facilitator Guide also pulled up either on a separate screen or in a separate window. It can be helpful to utilize a computer with a large monitor or two screens for this purpose. You will scroll through both of these during the course. An alternative option is to request a learner share their Learner Guide on the screen and scroll as needed.
5. Begin with Case 1 and request a learner read the non-bolded introductory information. Then, address each question to the subsequent learner, going in order around the group. Each learner will attempt to answer the question and, if unable to, the group can discuss to find a response together. If the question contains multiple parts such as with a broad differential, the facilitator can split it up, i.e., have each learner name one possible diagnosis. When learners are unable to respond, facilitator should help guide them to the correct answer.
6. Continue through Case 2 and Case 3 in the same manner. Offer the group a 5-minute break in between cases. Case 1 tends to be the longest and takes just over 30 minutes, case 2 takes around 30 minutes, and case 3 tends to be the shortest and takes just under 30 minutes.
7. Allow learners 10 minutes to complete the post-survey.
8. Have learners email their Learner Guide to you.
9. Email the Facilitator Guide to the learners following the curriculum to use for reference.
10. Option to send the delayed post-survey 4-8 weeks following the completion of the curriculum.

**CASE 1 (about 30 minutes)**

Your patient is a 32 year old presenting with chief complaint: “trouble getting pregnant, we have been trying for a year.”

The patient reports she started on birth control pills for dysmenorrhea as a teenager. She stopped these one year ago to attempt conception. She reports since stopping pills, she has had irregular cycles, every 2-3 months. Menses lasts 5-7 days, moderate flow. She has been having trouble timing intercourse due to this irregularity.

Her intake form has the following history:
OB history: Never pregnant

GYN history: Normal PAP/HPV co-test one year ago. Chlamydia at age 16, treated, negative test of cure.

Medical history: None

Surgical history: Appendectomy at age 12, not ruptured.

Family history: Adopted, unknown

Social: Denies tobacco/alcohol/drug use, works as a teacher. Cisgender male partner.

LMP: 13 weeks ago

**What review of systems questions would you ask?**

You perform the following review of systems:

Negative for hirsutism, deep cystic jawline acne, hair loss, unexplained weight gain or loss, temperature changes, galactorrhea, tunnel vision

**Write out PALM-COEIN. Which category do you think she falls into?**

PALM (Polyps, Adenomyosis, Leiomyoma, Malignancy) – Structural

COEIN (Coagulopathy, Ovulatory dysfunction, Endometrial, Iatrogenic, Not yet classified) – Nonstructural

Presenting with irregular menses/abnormal uterine bleeding, and based on PALM-COEIN with irregular cycles, unlikely to be a structural cause. Differential becomes more targeted to ovulatory dysfunction.^1^

**Considering reasons for infertility within this category, what is highest on your differential?**

Highest on your differential may be: polycystic ovarian syndrome (PCOS), primary ovarian insufficiency (POI), functional hypothalamic amenorrhea.

**What would you like to order to better evaluate her abnormal bleeding pattern and infertility?**

**Labs:**

**Imaging:**

It would be reasonable to start a complete infertility evaluation here. You could also begin with a targeted, staged assessment.

A correct response might include:

Labs: HCG (urine or serum), Anti-Mullerian Hormone (AMH), Follicle-stimulating hormone (FSH) and estradiol (E2) (Cycle day 2-4, but if your patient amenorrheic may need to induce menses with progestin withdrawal); prolactin, thyroid stimulating hormone (TSH), hemoglobin A1c (HbA1c)

Imaging: Hysterosalpingogram

It would not be wrong to also order:

Imaging: pelvic ultrasound

Other: Semen analysis for partner, progestin withdrawal as needed

Labs: Testosterone, 17-hydroxyprogesterone, DHEA-S, preconception labs

**You order and receive the following results:**

**E2 25 pg/mL**

**FSH 42 mIU/mL**

**AMH <0.01 ng/mL**

**Prolactin 12 ng/mL**

**TSH 2.3 mIU/L**

**Hgb A1c 4.9%**

**HCG negative**

**How do you interpret this?**

Her labs are concerning for primary ovarian insufficiency (previously known as premature ovarian failure or insufficiency). A normal follicle stimulating hormone (FSH) would be <10, and higher values can be suggestive of diminished ovarian reserve/premature aging.^2^ The definition of primary ovarian insufficiency (POI) is at least 3 months of menstrual irregularity with FSH >40-50 mIU/mL on two values one month apart, so you’ll need to plan a repeat FSH for this patient.

This diagnosis is not as rare as you might think – POI can make up 2-10% of cases of amenorrhea.

While AMH is not currently part of the diagnosis for POI, it can be helpful to raise suspicion for impaired ovarian reserve in a patient who may not meet diagnostic criteria yet, and potentially could capture a patient with diminished ovarian reserve when more fertility options are available to her. If she is planning fertility treatments, an AMH may also be helpful for dosing medications.

**What if the E2 had been 250 and FSH was 8?**

An elevated estradiol can suppress the FSH so that it can be lower or even in the normal range. This could be due to spontaneous ovulation in a patient with POI. In addition, in patients with diminished ovarian reserve/early ovarian aging, shortened cycles can lead to an early rise in estradiol that can suppress FSH, so they can have high estradiol and misleadingly normal FSH, even early in the menstrual cycle. If the estradiol is >60, a normal range FSH would not be considered reassuring, and further evaluation would still be indicated.

An elevated estradiol could also mean the patient was not on cycle day 2-4.

**Draw the HPO axis to remind yourself why FSH is so high.**

Hypothalamus

**GnRH +**

Pituitary

**-- Estradiol, progesterone**

**-- Inhibin**

**FSH and LH +**

Ovary

Author owned.

Inadequate negative feedback from the ovaries due to ovarian insufficiency.

**What are the possible causes of primary ovarian insufficiency, and based on this, what additional testing/evaluation do you order?**

Genetic causes:

Turner’s syndrome: although more commonly found in a case of primary amenorrhea or in a woman <age 30, patients can present later with varying degrees of Turner’s mosaicism. Referral to Genetic Counseling for karyotyping.

Fragile X syndrome: premutation in the FMR1 gene. Patients should be asked about specific family history of intellectual disability. Referral to Genetic Counseling for fragile X carrier premutation testing.

Iatrogenic, such as chemotherapy or radiation or pelvic surgery: Not consistent with this patient’s history.

There are multiple other genetic causes both known and unknown.^3,4^

Autoimmune:

Can be an autoimmune etiology, and combined with concomitant autoimmune conditions. Common autoimmune causes include autoimmune polyglandular syndrome, myasthenia gravis, and systemic lupus erythematous. Recommend testing for thyroid dysfunction (already completed) and adding thyroid auto-antibodies would also be reasonable. In addition, anti-adrenal antibody testing is recommended, as a positive result can portend a 50% risk of developing adrenal insufficiency. If positive, could consider referral to endocrinology, close monitoring clinically for signs/symptoms of adrenal insufficiency, and an annual corticotropin stimulation test.^3^

**You order the following evaluation:**

**Genetic counseling- 46XX karyotype, negative for FMR1 premutation**

**Anti-adrenal antibodies negative**

**You also repeat an E2/FSH one month later, and E2 is 32 with FSH 44, confirming the diagnosis.**

**How would you counsel this patient regarding fertility?**

Fertility: poor prognosis, however, women may still ovulate spontaneously and 5% may conceive.

**What treatment options are available?**

If she not interested in pregnancy, recommend use of contraception, either hormonal or barrier methods, as patients may still occasionally ovulate and conceive.

If she is interested in pregnancy, you can recommend a lower dose of hormonal therapy to allow for potential spontaneous ovulation. In this case, some providers will opt for dosing with cyclic bleeding, as this could help with early recognition of a pregnancy and also allows for a period of time with endometrial growth that could facilitate implantation.

Donor egg or donor embryo, as well as adoption, should be discussed as options for family building.

Use of ovulation induction with clomiphene citrate and letrozole, while not harmful, are considered futile in this patient population. In addition, this patient would not respond to stimulation medications for in vitro fertilization. Her FSH is already very high, so adding more FSH would not make her ovaries respond.

**How would you counsel this patient regarding general health and well-being, and what additional testing might you order?**

At risk for complications of estrogen deficiency: bone loss and fracture, cardiovascular disease, vasomotor symptoms, decline in cognitive function, to name a few. Recommend a baseline dual energy X-ray absorptiometry (DEXA) scan at time of diagnosis, and use of hormonal therapy in this population is essential. In addition, psychosocial support cannot be understated, and a referral to a mental health provider should be offered.

**What hormonal therapy would you order for this patient?**

There is a helpful table in the ACOG practice bulletin on primary ovarian insufficiency for hormonal dosages of estrogen and progestogen, both continuous and sequential.^4^ In addition, hormonal contraceptives can be used if the patient is not planning a pregnancy. Hormonal therapy should be continued until at least age 50-51.

**CASE 2 (about 30 minutes)**

Your patient is a 28 year old presenting with difficulty getting pregnant for 4 months and the chief complaint, “I’m trying to use ovulation predictor kits, but they just never turn positive for me.”

The patient reports she had heavy and irregular periods as a teenager. They became regular for a few years in her mid-20s, but have become increasingly irregular over the past year. She shows you her menstrual cycle app which shows cycles ranges from 20-60 days. She tells you her periods are sometimes very heavy requiring multiple boxes of tampons, but other times so light she only needs a pantiliner.

Her intake form has the following history:
OB history: Termination via D&C at age 19

GYN history: Abnormal PAP at 22, but normal PAPs since

Medical history: BMI 32

Surgical history: None

Family history: Mother had trouble getting pregnant and had endometrial cancer at age 45, s/p hysterectomy

Social: Cisgender male partner. No tobacco, occasional marijuana and alcohol, works as a librarian.

**What targeted clinical questions would you ask?**

You may ask about signs of hyperandrogenism, hyperprolactinemia, risks for tubal factor. You could ask about moliminal symptoms, pelvic pain.

**Positive for hirsutism (“I’ve had increasing hair growth along my chin so now I need to pluck every other day”), and some thinning of hair at hairline. Denies unexplained weight gain or loss, temperature changes, galactorrhea or tunnel vision. No history of sexually transmitted infection.**

**LMP: 58 days ago**

**What common condition is highest on your differential?**

Diagnosis is irregular menses/abnormal uterine bleeding, and based on PALM-COEIN with irregular cycles, unlikely to be a structural cause. Again, differential becomes more targeted to ovulatory dysfunction.^1^

PCOS may be highest on your differential.

Other causes of ovulatory dysfunction, idiopathic hirsutism would also be reasonable.

**What less common conditions are also on your differential diagnosis?**

- Nonclassic congenital adrenal hyperplasia
- Virilizing adrenal or ovarian tumor
- Cushing’s syndrome

**What would you like to order to better evaluate her abnormal bleeding pattern and infertility?**

**Labs:**

**Imaging:**

It would be reasonable to start a complete infertility evaluation here. You could also begin with a targeted assessment.

A correct response might include:

Labs: HCG (urine or serum), follicle-stimulating hormone (FSH) and estradiol (E2) (ideally cycle day 2-4; could induce menses with a progestin withdrawal); total testosterone, 17-hydroxyprogesterone (17-OHP), progesterone, dehydroepiandrosterone sulfate (DHEA-S), prolactin, thyroid stimulating hormone (TSH), hemoglobin A1c, lipids.

Imaging: Hysterosalpingogram

Other: Semen analysis

It would not be wrong to also order:

Labs: Anti-Mullerian hormone (AMH), mid-luteal progesterone

Imaging: Additional uterine imaging, a pelvic ultrasound

**When should these labs be drawn?**

FSH and E2, on cycle day 3 (or, 2-4) as discussed in case 1. Both testosterone and 17-OHP should also be drawn in the early follicular phase, ideally in the morning. After ovulation, with the rise in progesterone, 17-OHP can be elevated. If you’re not sure where you are in the cycle (like in this patient with irregular cycles), you should order a progesterone level to help you interpret the 17-OHP.

You may have heard the term “day 21 progesterone” to confirm ovulation. However, this will only be useful in a ~28 day cycle. The goal is to draw your progesterone in the mid-luteal phase, or 7 days prior to when menses is expected. A value > 3.0 ng/mL is consistent with having ovulated.

If possible, prolactin should be drawn first thing in morning after no nipple stimulation or sexual activity for three days. If this is not possible, it is reasonable to get a random prolactin level and repeat with these restrictions only if it is elevated.

Depending on how irregular your patient’s menses are, you could induce menses with a progestin withdrawal to help to time labs.

**You order and receive the following results:**

**FSH, E2, 17-OHP, prolactin, TSH, Hemoglobin A1c, HCG, total testosterone, DHEA-S, HSG, semen analysis are all normal range, urine HCG is negative, AMH is slightly elevated**

**How do you interpret this? What is the most likely diagnosis?**

History of infertility with irregular cycles and hirsutism is most likely polycystic ovarian syndrome, after ruling out other potential causes.

**What are the Rotterdam criteria for diagnosis of PCOS?**

Exclusion of others causes of oligoovulation and at least 2/3 of the following:

1. Oligoovulation or anovulation
2. Clinical or biochemical signs of hyperandrogenism
3. Polycystic ovaries on ultrasound (at least one ovary with ≥12-20 follicles measuring 2-9mm in diameter and/or volume >10 mL)

**What is on the full differential for hirsutism in an adult woman?**

1. PCOS
2. Nonclassical congenital adrenal hyperplasia (NC-CAH)
3. Androgen secreting ovarian or adrenal tumor
4. Hyperandrogenism, insulin resistance, and acanthosis nigricans (HAIR-AN) syndrome
5. Ovarian hyperthecosis
6. Cushing’s syndrome including an ACTH secreting tumor
7. Exogenous testosterone exposure (like a partner’s androgen gel on a hand towel)
8. Growth hormone secreting tumor

**For the above conditions, what testing do you get to rule out the more common conditions besides PCOS?**

Idiopathic hirsutism: normal menses and normal range serum androgens. (Diagnosis of exclusion, after excluding below conditions)

Nonclassical congenital adrenal hyperplasia: most commonly 21-hydroxylase deficiency, which is suggested by elevated 17-OHP >200ng/dL (6 nmol/L), and diagnosis is confirmed with ACTH stimulation test.

The conditions below usually have more impressive virilization or lab abnormalities than this patient describes:

Androgen-secreting tumor of ovary or adrenals: Serum testosterone >150-200 ng/dL, or DHEAS >700 ng/dl, imaging (TVUS and CT, respectively).

Hyperandrogenism, insulin-resistant, and acanthosis nigricans (HAIR-AN) syndrome: More severe clinical features than PCOS. Testosterone levels, specifically free testosterone, can be quite high.

Ovarian hyperthecosis: ultrasound imaging may show increased ovarian size but without multicystic appearance; clinical features include significant virilization that progresses slowly and features of metabolic syndrome, with total testosterone >150 ng/dL.

**You make the diagnosis of PCOS.** **What would you offer her for fertility treatment?**

Since she has irregular cycles, she likely has anovulation resulting from PCOS. The first line fertility treatment for PCOS is ovulation induction with letrozole.

Historically clomiphene citrate was first line oral medication for all ovulation induction. The PPCOS II randomized control trial supported letrozole as superior for PCOS patients. It also appeared to have similar safety of conceived pregnancies compared to clomiphene citrate.^5^ Of note, letrozole is not FDA approved for ovulation induction, despite reassuring studies, whereas clomiphene citrate is.

**What is the mechanism of action of letrozole?**

Letrozole is an aromatase inhibitor. Aromatase (also referred to as CYP19A1) converts androgens into estrogens. By blocking aromatase, estradiol does not increase as significantly throughout the follicular phase, removing negative feedback at the hypothalamus and pituitary, and leading to increased FSH secretion.

Testosterone

Androstenedione

Letrozole

Aromatase (CYP191A1)

Estradiol (E2)

Estrone (E1)

Author owned.

**How should you order letrozole for your patient? What is the dose and how is it taken?**

The starting dose is 2.5 mg, and you can increase it by 2.5 mg each cycle to a maximum dose of 7.5 mg. It is administered orally for a 5-day course, typically starting cycle days 3 (some providers may start as late as days 4 or 5). Pregnancy should be excluded before starting letrozole (as it has been in this patient). If they’re amenorrheic, you may begin the letrozole without inducing menses, but many providers start with a progestin withdrawal to induce menses.

**How do you monitor whether she ovulated or not?**

Your patient can use ovulation predictor kits (OPKs) to confirm ovulation and help time intercourse. OPKs measure LH in the urine. Ovulation occurs approximately 36-48 hours after the onset of the LH surge in the blood. Since LH is detectable in the urine approximately 12 hours after onset of the LH surge, the patient should anticipate ovulating the day after she has a positive OPK. It is best if the sperm is already there at the time of ovulation, as sperm can live for several days in the reproductive tract. Intercourse on the day of the surge and the next day is a reasonable recommendation. However, false positive LH surges are common in PCOS patients, due to high basal LH. You can confirm ovulation occurred with a progesterone level 7 days later.^6^

If you have access to transvaginal ultrasound, you can perform monitoring with an ultrasound, usually around cycle day 12, to confirm the development of at least one ovulatory follicle (a hypoechoic ovarian cyst typically >16mm). Some providers will use an hCG trigger injection to induce ovulation when a large follicle is noted (>18-22mm), to help time either intercourse or intrauterine insemination.

**What else should you counsel your patient on if you are prescribing letrozole?**

You should counsel your patient on a 3-7% risk of multiple pregnancy. Most cases of multiples are twins, but rarely, higher order multiples can occur.^6^

**For a patient with a new PCOS diagnosis, what other evaluation and counseling are important to address?**

The patient should be evaluated for the features of metabolic syndrome. This is detailed in the ACOG practice bulletin on PCOS, but includes evaluation of blood pressure, body mass index, lipids, and insulin resistance. Patient should also be counseled on risk of persistent anovulation on developing a uterine malignancy and recommendation for progestin therapy when not attempting conception.^7^

**CASE 3 (about 30 minutes)**

You are covering GYN consults overnight in a community hospital. You receive a call from the ED about a patient they are actively triaging and concerned about her acuity.

The patient is a 23 year old who had an oocyte retrieval this morning following IVF to serve as an egg donor at a private clinic. You don’t have access to her records. She told the ED, “I think they got 30 or 40 eggs?” She reports no significant past medical or surgical history. She does report painful menses at baseline.

She presented to the ED this evening with nausea, increasing abdominal pain and distension and shortness of breath. She vomited after trying to take her pain medication.

In the emergency department, her vitals are the following: HR 120, BP 90/60, RR 22. Focused assessment with sonography for trauma (FAST) scan is positive for fluid throughout the abdomen. The ED is concerned about blood loss and would like to start a blood transfusion and for you to consider taking her to the operating room expeditiously.

**What is on your differential?**

Ovarian hyperstimulation syndrome (OHSS)

Venous thromboembolism

Post-retrieval hemorrhage

Bowel or bladder injury

Ovarian torsion

Tubo-ovarian abscess- has dysmenorrhea, and if she had endometriomas, this would be a risk factor; however, unlikely to present on the same day of the procedure.

Complications after oocyte retrieval are rare (not including OHSS) at <1%, and risk of requiring a surgical intervention is 1/1000.

Following an oocyte retrieval in a highly responsive patient, you should generally wait for a complete blood count or CBC (at least a blood gas hemoglobin), even in a somewhat unstable patient, as hypotension, abdominal distention/pain, and a positive FAST scan can be signs of both OHSS and acute blood loss. You can assess a patient’s degree of response and their risk for OHSS just by asking “how many eggs were retrieved?”, which most patients will know. If >30 oocytes were retrieved, risk of OHSS is significantly higher.

**What are risk factors for post-retrieval complications?**

Risk factors for post-retrieval complications include patients who are younger, thinner, and have high number of oocytes retrieved.

**Her labs return and are notable for Hgb 6.5 and Hct 19.0. Other labs are normal. What are you worried about, and what are your next actions?**

Her labs are concerning for post-retrieval bleeding. You would manage this similar to other post-operative bleeding patients. You should start by assessing and ensuring hemodynamic stability and identifying the likely source of bleeding.

Depending on stability and exam, next steps could also include:
Labs: Coagulopathy panel, trend CBCs

Imaging: FAST scan in emergency department if not already completed, pelvic ultrasound, computerized tomography (CT) scan

Interventions: Serial abdominal exams, IV fluids, pressor support (managed with emergency medicine or intensive care unit), blood product transfusion, interventional radiology embolization, laparoscopy (or rarely, laparotomy)

**What are the possible causes/locations of acute blood loss following oocyte retrieval?**

An egg retrieval occurs by placing an ultrasound guided needle through the vaginal wall and into each ovary.

A post-retrieval bleed can be caused by damage to ovarian or vaginal vessels, or rarely, bleeding from injury to surrounding organs. Usually a bleed would present within the first day, but could occur as late as a week after, due to multiple hemorrhagic corpus lutea. Major vessel injuries can also occur due to the proximity of the internal iliac artery and vein. Bleeding may be intraperitoneal, vaginal, or retroperitoneal.^8^

An exam is warranted, and if present, vaginal bleeding would usually be apparent. Vaginal bleeding is typically at the needle site. If vaginal bleeding is encountered, it can be managed with pressure, hemostatic agents, or a suture at site of bleeding. If bleeding persists, care should be taken when placing hemostatic stitches not to risk ureteral or bladder injury.

If intraperitoneal bleeding is suspected, surgical intervention may be indicated if patient cannot be adequately stabilized. Interventional Radiology consultation can be considered as well. Keep in mind that it may be difficult to identify the source of bleeding surgically as the injury was caused by a large (such as a 16 gauge) needle. While fertility preservation should be optimized, patients should be consented for possible need for oophorectomy if bleeding is identified from the ovarian vessels or on the ovary itself, and cannot be controlled with other measures.

It is also important to remember that even mild OHSS can lead to some hemoconcentration of lab values, so a slightly low or even low-normal Hemoglobin may be falsely elevated, and a high index of suspicion should be maintained.

**Let’s track back and change the scenario. The patient is reporting the above symptoms, as well as low and concentrated urine output. Now her labs resulted and are notable for:
Hgb 14 and Hct 42 %, WBC 17, Cr 1.7. FAST exam shows ascites.**

**Have you changed your most likely diagnosis?**

Ovarian hyperstimulation syndrome (OHSS) is the most likely diagnosis. Lab findings consistent with OHSS include hemoconcentration, electrolyte imbalances, and renal injury.

The ASRM guideline on OHSS has a table that defines mild, moderate, severe, and critical OHSS.^9^ This patient meets lab and clinical criteria for severe OHSS due to her Cr >1.7.

**What is your next step in management?**

Recommend inpatient admission, and chest X-ray if patient has shortness of breath. Patient’s with OHSS are intravascularly depleted but have significant third spacing. Serious complications of OHSS can include: venous thrombosis, renal failure, thromboembolism, and adult respiratory distress syndrome, among others. Management of OHSS is complex and usually involves a combination of paracentesis or culdocentesis and intravenous fluids. Thromboprophylaxis is recommended. There is mixed evidence on use of volume expanders and/or diuretics.

**What might your admission orders be for a patient with this condition?**

Daily weight

Strict ins and outs

Out of bed to bathroom

Regular diet

Telemetry if hyperkalemic

CBC, coagulopathy panel, complete metabolic panel

Prophylactic anticoagulation, such as low molecular weight heparin and use of sequential compression devices

Intravenous fluids

**What were the patient’s risk factors for this diagnosis?**

Younger patients with high response to ovarian stimulation, such as oocyte donors or patients with PCOS, are at higher risk for OHSS. Luckily, there have been changes in IVF protocols and practice, such as the use of gonadotropin-releasing hormone agonist (leuprolide) trigger and freeze-all cycles, that have reduced hospitalizations for OHSS, but it should be promptly recognized and treated.

References

1. American College of Obstetricians and Gynecologists' Committee on Practice Bulletins—Gynecology. ACOG Practice Bulletin No. 128: diagnosis of abnormal uterine bleeding in reproductive-aged women. *Obstet Gynecol*. 2012;120(1):197-206. doi:10.1097/AOG.0b013e318262e320

2. American College of Obstetricians and Gynecologists' Committee on Adolescent Health Care. ACOG Committee Opinion No. 605: primary ovarian insufficiency in adolescents and young women. *Obstet Gynecol*. 2014;124(1):193-197. doi:10.1097/01.AOG.0000451757.51964.98

3. Nelson LM. Clinical Practice. Primary Ovarian Insufficiency. *N Engl J Med*. 2009;360(6):606-614. doi:10.1056/NEJMcp0808697

4. Taylor HS, Lubna P, Seli E. *Speroff’s Clinical Gynecologic Endocrinology and Infertility*. 9th edition. Wolters Kluwer; 2020. Chapters 11, 12, and 27.

5. Legro RS, Brzyski RG, Diamond MP, et al. Letrozole versus Clomiphene for Infertility in the Polycystic Ovary Syndrome. *N Engl J Med*. 2014;371(2):119-129. doi:10.1056/NEJMoa1313517

6. American College of Obstetricians and Gynecologists' Committee on Practice Bulletins—Gynecology. ACOG Practice Bulletin No. 194: Polycystic Ovary Syndrome [published correction appears in Obstet Gynecol. 2020 Sep;136(3):638]. *Obstet Gynecol*. 2018;131(6):e157-e171. doi:10.1097/AOG.0000000000002656

7. Levi-Setti PE, Cirillo F, Scolaro V, et al. Appraisal of clinical complications after 23,827 oocyte retrievals in a large assisted reproductive technology program. *Fertil Steril*. 2018;109(6):1038-1043.e1. doi:10.1016/j.fertnstert.2018.02.002

8. Prevention and treatment of moderate and severe ovarian hyperstimulation syndrome: a guideline. *Fertility and Sterility*. 2016;106(7):1634-1647. doi:10.1016/j.fertnstert.2016.08.048

**Answers for pre- and post-survey multiple choice questions (underlined and bolded):**

Knowledge assessment:

Your patient is a 32 year old with polycystic ovarian syndrome interested in fertility treatment. She only has a few menstrual cycles a year at her baseline. The starting dose of letrozole for ovulation induction is:

1. 100 mcg, oral, for 3 days
2. 25 mg, oral, for 3 days
3. **2.5 mg, oral, for 5 days**
4. 10 mcg, oral, for 5 days

Your 28 year old patient is considering donating her eggs to a sister who recently underwent chemotherapy. She asks about the risks of egg donation, specifically, ovarian hyperstimulation syndrome (OHSS). Severe complications of OHSS include all of the following **EXCEPT:**

1. Pulmonary embolism
2. Renal failure
3. Acute respiratory distress syndrome
4. **Anemia requiring transfusion**

Your 26 year old patient reports that her brother has fragile X syndrome. She is not sure if she is a fragile X premutation carrier, but asks if she were, whether it would pose any health risks for her. Fragile X premutation is associated with:

1. **Primary ovarian insufficiency**
2. Recurrent pregnancy loss
3. Mullerian agenesis
4. Isolated teratospermia
