# Supplementary material for: Strengthening the Reproductive Endocrinology and Infertility Curriculum Through Three Interactive Cases
Source: MedEdPORTAL. 2023 Dec 21;19:11375. doi: 10.15766/mep_2374-8265.11375 (PMC10733559; doi:10.15766/mep_2374-8265.11375)
Supplement: Supplementary file 1 — Learner Guide and Pre- and Postsurveys.docxFacilitator Guide.docxDelayed Postsurvey.docx [file mep_2374-8265.11375-s001.zip › C. Delayed Postsurvey.docx]

**Delayed Post-Test/Survey** (Administered 4-8 weeks post-intervention)

How do you feel your knowledge and competence in the field of reproductive endocrinology and infertility compares to the other subspecialties in OB/GYN?

Knowledge:

1 2 3 4 5

Significantly worse Same Significantly better

Competence:

1 2 3 4 5

Significantly worse Same Significantly better

Your patient is a 32 year old with polycystic ovarian syndrome interested in fertility treatment. She only has a few menstrual cycles a year at her baseline. The starting dose of letrozole for ovulation induction is:

A. 100 mcg, oral, for 3 days

B. 25 mg, oral, for 3 days

C. 2.5 mg, oral, for 5 days

D. 10 mcg, oral, for 5 days

Your patient is considering donating her eggs to a sister who recently underwent chemotherapy. She asks about the risks of egg donation, specifically, ovarian hyperstimulation syndrome (OHSS). Severe complications of OHSS include all of the following **EXCEPT:**

A. Pulmonary embolism

B. Renal failure

C. Acute respiratory distress syndrome

D. Anemia requiring transfusion

Your 26 year old patient reports that her brother has fragile X syndrome. She is not sure if she is a fragile X premutation carrier, but asks if she were, whether it would pose any health risks for her. Fragile X premutation is associated with:

A. Primary ovarian insufficiency

B. Recurrent pregnancy loss

C. Mullerian agenesis

D. Isolated teratospermia
