# Supplementary material for: Strengthening the Reproductive Endocrinology and Infertility Curriculum Through Three Interactive Cases
Source: MedEdPORTAL. 2023 Dec 21;19:11375. doi: 10.15766/mep_2374-8265.11375 (PMC10733559; doi:10.15766/mep_2374-8265.11375)
Supplement: Supplementary file 1 — Learner Guide and Pre- and Postsurveys.docxFacilitator Guide.docxDelayed Postsurvey.docx [file mep_2374-8265.11375-s001.zip › A. Learner Guide and Pre- and Postsurveys.docx]

**Pre-Survey (10 minutes)**

What PGY year are you?

1 2 3 4 Other (non-resident):

*The following questions are to gain understanding of your comfort level in clinical practice. This can be when you actually see a patient, or, if you’ve never seen a patient with these concerns, theoretically if you were to see a patient with this concern.*

How comfortable do you feel evaluating and managing patients with the chief complaint of irregular menses?

Evaluating:

1 2 3 4 5

Not comfortable at all Neutral Very comfortable

Managing:

1 2 3 4 5

Not comfortable at all Neutral Very comfortable

How comfortable do you feel evaluating and managing patients with the chief complaint of infertility?

Evaluating:

1 2 3 4 5

Not comfortable at all Neutral Very comfortable

Managing:

1 2 3 4 5

Not comfortable at all Neutral Very comfortable

How comfortable do you feel evaluating and managing patients with the primary complaint of secondary amenorrhea?

Evaluating:

1 2 3 4 5

Not comfortable at all Neutral Very comfortable

Managing:

1 2 3 4 5

Not comfortable at all Neutral Very comfortable

How do you feel your knowledge and competence in the field of reproductive endocrinology and infertility compares to the other subspecialties in OB/GYN?

Knowledge:

1 2 3 4 5

Significantly worse Same Significantly better

Competence:

1 2 3 4 5

Significantly worse Same Significantly better

Knowledge assessment:

Your patient is a 32 year old with polycystic ovarian syndrome interested in fertility treatment. She only has a few menstrual cycles a year at her baseline. The starting dose of letrozole for ovulation induction is:

1. 100 mcg, oral, for 3 days
2. 25 mg, oral, for 3 days
3. 2.5 mg, oral, for 5 days
4. 10 mcg, oral, for 5 days

Your 28 year old patient is considering donating her eggs to a sister who recently underwent chemotherapy. She asks about the risks of egg donation, specifically, ovarian hyperstimulation syndrome (OHSS). Severe complications of OHSS include all of the following **EXCEPT:**

1. Pulmonary embolism
2. Renal failure
3. Acute respiratory distress syndrome
4. Anemia requiring transfusion

Your 26 year old patient reports that her brother has fragile X syndrome. She is not sure if she is a fragile X premutation carrier, but asks if she were, whether it would pose any health risks for her. Fragile X premutation is associated with:

1. Primary ovarian insufficiency
2. Recurrent pregnancy loss
3. Mullerian agenesis
4. Isolated teratospermia

**CASE 1 (about 30 minutes)**

Your patient is a 32 year old presenting with chief complaint: “trouble getting pregnant, we have been trying for a year.”

The patient reports she started on birth control pills for dysmenorrhea as a teenager. She stopped these one year ago to attempt conception. She reports since stopping pills, she has had irregular cycles, every 2-3 months. Menses lasts 5-7 days, moderate flow. She has been having trouble timing intercourse due to this irregularity.

Her intake form has the following history:
OB history: Never pregnant

GYN history: Normal PAP/HPV co-test one year ago. Chlamydia at age 16, treated, negative test of cure.

Medical history: None

Surgical history: Appendectomy at age 12, not ruptured.

Family history: Adopted, unknown

Social: Denies tobacco/alcohol/drug use, works as a teacher. Cisgender male partner.

LMP: 13 weeks ago

**What review of systems questions would you ask?**

**Write out PALM-COEIN. Which category do you think she falls into?**

**Considering reasons for infertility within this category, what is highest on your differential?**

**What would you like to order to better evaluate her abnormal bleeding pattern and infertility?**

**Labs:**

**Imaging:**

**You order and receive the following results:**

**E2 25 pg/mL**

**FSH 42 mIU/mL**

**AMH <0.01 ng/mL**

**Prolactin 12 ng/mL**

**TSH 2.3 mIU/L**

**Hgb A1c 4.9%**

**HCG negative**

**How do you interpret this?**

**What if the E2 had been 250 and FSH was 8?**

**Draw the HPO axis to remind yourself why FSH is so high.**

**What are the possible causes of primary ovarian insufficiency, and based on this, what additional testing/evaluation do you order?**

**You order the following evaluation:**

**Genetic counseling- 46XX karyotype, negative for FMR1 premutation**

**Anti-adrenal antibodies negative**

**You also repeat an E2/FSH one month later, and E2 is 32 with FSH 44, confirming the diagnosis.**

**How would you counsel this patient regarding fertility?**

**What treatment options are available?**

**How would you counsel this patient regarding general health and well-being, and what additional testing might you order?**

**What hormonal therapy would you order for this patient?**

**CASE 2 (about 30 minutes)**

Your patient is a 28 year old presenting with difficulty getting pregnant for 4 months and the chief complaint, “I’m trying to use ovulation predictor kits, but they just never turn positive for me.”

The patient reports she had heavy and irregular periods as a teenager. They became regular for a few years in her mid-20s, but have become increasingly irregular over the past year. She shows you her menstrual cycle app which shows cycles ranges from 20-60 days. She tells you her periods are sometimes very heavy requiring multiple boxes of tampons, but other times so light she only needs a pantiliner.

Her intake form has the following history:
OB history: Termination via D&C at age 19

GYN history: Abnormal PAP at 22, but normal PAPs since

Medical history: BMI 32

Surgical history: None

Family history: Mother had trouble getting pregnant and had endometrial cancer at age 45, s/p hysterectomy

Social: Cisgender male partner. No tobacco, occasional marijuana and alcohol, works as a librarian.

**What targeted clinical questions would you ask?**

**Positive for hirsutism (“I’ve had increasing hair growth along my chin so now I need to pluck every other day”), and some thinning of hair at hairline. Denies unexplained weight gain or loss, temperature changes, galactorrhea or tunnel vision. No history of sexually transmitted infection.**

**LMP: 58 days ago**

**What common condition is highest on your differential?**

**What less common conditions are also on your differential diagnosis?**

**What would you like to order to better evaluate her abnormal bleeding pattern and infertility?**

**Labs:**

**Imaging:**

**When should these labs be drawn?**

**You order and receive the following results:**

**FSH, E2, 17-OHP, prolactin, TSH, Hemoglobin A1c, HCG, total testosterone, DHEA-S, HSG, semen analysis are all normal range, urine HCG is negative, AMH is slightly elevated**

**How do you interpret this? What is the most likely diagnosis?**

**What are the Rotterdam criteria for diagnosis of PCOS?**

**What is on the full differential for hirsutism in an adult woman?**

**For the above conditions, what testing do you get to rule out the more common conditions besides PCOS?**

**You make the diagnosis of PCOS.** **What would you offer her for fertility treatment?**

**What is the mechanism of action of letrozole?**

**How should you order letrozole for your patient? What is the dose and how is it taken?**

**How do you monitor whether she ovulated or not?**

**What else should you counsel your patient on if you are prescribing letrozole?**

**For a patient with a new PCOS diagnosis, what other evaluation and counseling are important to address?**

**CASE 3 (about 30 minutes)**

You are covering GYN consults overnight in a community hospital. You receive a call from the ED about a patient they are actively triaging and concerned about her acuity.

The patient is a 23 year old who had an oocyte retrieval this morning following IVF to serve as an egg donor at a private clinic. You don’t have access to her records. She told the ED, “I think they got 30 or 40 eggs?” She reports no significant past medical or surgical history. She does report painful menses at baseline.

She presented to the ED this evening with nausea, increasing abdominal pain and distension and shortness of breath. She vomited after trying to take her pain medication.

In the emergency department, her vitals are the following: HR 120, BP 90/60, RR 22. Fast scan is positive for fluid throughout the abdomen. The ED is concerned about blood loss and would like to start a blood transfusion and for you to consider taking her to the operating room expeditiously.

**What is on your differential?**

**What are risk factors for post-retrieval complications?**

**Her labs return and are notable for Hgb 6.5 and Hct 19.0. Other labs are normal. What are you worried about, and what are your next actions?**

**What are the possible causes/locations of acute blood loss following oocyte retrieval?**

**Let’s track back and change the scenario. The patient is reporting the above symptoms, as well as low and concentrated urine output. Now her labs resulted and are notable for:
Hgb 14 and Hct 42 %, WBC 17, Cr 1.7. FAST exam shows ascites.**

**Have you changed your most likely diagnosis?**

**What is your next step in management?**

**What might your admission orders be for a patient with this condition?**

**What were the patient’s risk factors for this diagnosis?**

References

1. American College of Obstetricians and Gynecologists' Committee on Practice Bulletins—Gynecology. ACOG Practice Bulletin No. 128: diagnosis of abnormal uterine bleeding in reproductive-aged women. *Obstet Gynecol*. 2012;120(1):197-206. doi:10.1097/AOG.0b013e318262e320

2. American College of Obstetricians and Gynecologists' Committee on Adolescent Health Care. ACOG Committee Opinion No. 605: primary ovarian insufficiency in adolescents and young women. *Obstet Gynecol*. 2014;124(1):193-197. doi:10.1097/01.AOG.0000451757.51964.98

3. Nelson LM. Clinical Practice. Primary Ovarian Insufficiency. *N Engl J Med*. 2009;360(6):606-614. doi:10.1056/NEJMcp0808697

4. Taylor HS, Lubna P, Seli E. *Speroff’s Clinical Gynecologic Endocrinology and Infertility*. 9th edition. Wolters Kluwer; 2020. Chapters 11, 12, and 27.

5. Legro RS, Brzyski RG, Diamond MP, et al. Letrozole versus Clomiphene for Infertility in the Polycystic Ovary Syndrome. *N Engl J Med*. 2014;371(2):119-129. doi:10.1056/NEJMoa1313517

6. American College of Obstetricians and Gynecologists' Committee on Practice Bulletins—Gynecology. ACOG Practice Bulletin No. 194: Polycystic Ovary Syndrome [published correction appears in Obstet Gynecol. 2020 Sep;136(3):638]. *Obstet Gynecol*. 2018;131(6):e157-e171. doi:10.1097/AOG.0000000000002656

7. Levi-Setti PE, Cirillo F, Scolaro V, et al. Appraisal of clinical complications after 23,827 oocyte retrievals in a large assisted reproductive technology program. *Fertil Steril*. 2018;109(6):1038-1043.e1. doi:10.1016/j.fertnstert.2018.02.002

8. Prevention and treatment of moderate and severe ovarian hyperstimulation syndrome: a guideline. *Fertility and Sterility*. 2016;106(7):1634-1647. doi:10.1016/j.fertnstert.2016.08.048

**Post-Survey (10 minutes)**

How satisfied were you with this educational curriculum?

1 2 3 4 5

Not satisfied at all Extremely satisfied

How much did you enjoy participating in this educational curriculum?

1 2 3 4 5

Did not enjoy at all Enjoyed very much

How comfortable do you feel evaluating and managing patients with the chief complaint of irregular menses?

Evaluating:

1 2 3 4 5

Not comfortable at all Neutral Very comfortable

Managing:

1 2 3 4 5

Not comfortable at all Neutral Very comfortable

How comfortable do you feel evaluating and managing patients with the chief complaint of infertility?

Evaluating:

1 2 3 4 5

Not comfortable at all Neutral Very comfortable

Managing:

1 2 3 4 5

Not comfortable at all Neutral Very comfortable

How comfortable do you feel evaluating and managing patients with the primary complaint of secondary amenorrhea?

Evaluating:

1 2 3 4 5

Not comfortable at all Neutral Very comfortable

Managing:

1 2 3 4 5

Not comfortable at all Neutral Very comfortable

How do you feel your knowledge and competence in the field of reproductive endocrinology and infertility compares to the other subspecialties in OB/GYN?

Knowledge:

1 2 3 4 5

Significantly worse Same Significantly better

Competence:

1 2 3 4 5

Significantly worse Same Significantly better

Knowledge assessment:

The starting dose of letrozole for ovulation induction is:

1. 100 mcg, oral, for 3 days
2. 25 mg, oral, for 3 days
3. 2.5 mg, oral, for 5 days
4. 10 mcg, oral, for 5 days

Severe complications of ovarian hyperstimulation syndrome include all of the following **EXCEPT:**

1. Pulmonary embolism
2. Renal failure
3. Acute respiratory distress syndrome
4. Anemia requiring transfusion

Fragile X premutation is associated with:

1. Primary ovarian insufficiency
2. Recurrent pregnancy loss
3. Mullerian agenesis
4. Isolated teratospermia

Comments:

General feedback?

What do you like or dislike about this curriculum?

What did you learn that you did not know before?

Will this change your practice, and if so, how?
