# Supplementary material for: Strengthening the Reproductive Endocrinology and Infertility Curriculum Through Three Interactive Cases
Source: MedEdPORTAL. 2023 Dec 21;19:11375. doi: 10.15766/mep_2374-8265.11375 (PMC10733559; doi:10.15766/mep_2374-8265.11375)
Supplement: Supplementary file 1 — Learner Guide and Pre- and Postsurveys.docxFacilitator Guide.docxDelayed Postsurvey.docx [file mep_2374-8265.11375-s001.zip › _Educational Summary Report_11375.pdf]

MedEdPORTAL® The AAMC Journal of Teaching and Learning Resources

Original Publication Open Access

Strengthening the Reproductive Endocrinology and Infertility Curriculum Through Three Interactive Cases

Molly Siegel Kornfield, MD*, Elizabeth Rubin, MD, Pamela Parker, MD, MPH, Bharti Garg, MBBS, MPH, Amy Stenson, MD, MPH, Paula Amato, MD, Sacha Krieg, MD, PhD

*Corresponding author: mollyesiegel@gmail.com

Abstract

Introduction: Improved reproductive endocrinology and infertility (REI) curricula are needed to address educational deficiencies both at our institution and on a national level. To improve REI education for OB/GYN residents and medical students, we developed and piloted a curriculum with in-person and virtual flexibility. **Methods:** We developed three clinical vignettes for a facilitator-led case-based discussion among OB/GYN residents: two office cases and one emergency scenario. Cases were evaluated by content experts and tested before implementation. Pre- and postsurveys included both multiple-choice questions on content and a Likert-scale self-assessment of comfort, satisfaction, and knowledge. Postsurveys were administered immediately postintervention and at a delayed interval. Responses were compared using paired *t* tests and McNemar tests. **Results:** Eighteen learners (16 OB/GYN residents and two medical students) participated, the majority in person, of whom 17 (94%) completed a postsurvey. Self-rated proficiency in evaluating and managing irregular menses, infertility, and amenorrhea all improved significantly immediately following the intervention ($p < .05$ for all). Learners reported significantly more knowledge and comfort with REI compared to other subspecialties following the intervention ($p < .05$). More learners responded correctly to knowledge questions postintervention ($p < .05$ for questions 1 and 2, $p = .16$ for question 3). All learners were satisfied with and enjoyed the curriculum. Eight learners completed the delayed postsurvey and showed sustained improvements in knowledge and competence with REI content. **Discussion:** Facilitator-guided case-based learning was effective in improving learners' confidence, comfort, and knowledge in managing REI conditions, and improvements were sustained following a delayed interval.

Keywords
Amenorrhea, Egg Retrieval, Fertility, Infertility, Reproductive Endocrinology, Case-Based Learning, OB/GYN - Reproductive Endocrinology/Infertility, Problem-Based Learning,

Educational Objectives

By the end of this activity, learners will be able to:

1. Describe the initial evaluation of secondary amenorrhea, develop and prioritize a differential diagnosis, and interpret diagnostic results to identify the appropriate likely etiology.
2. Select the appropriate treatments, with regard to both fertility treatments and general medical care, for two of the most common causes of secondary amenorrhea.
3. Describe the possible complications that can occur following an oocyte retrieval and risk factors for these complications, interpret diagnostic findings to identify the appropriate likely etiology, and outline appropriate management of two common complications that can occur following an oocyte retrieval.

Citation:
Kornfield MS, Rubin E, Parker P, et al. Strengthening the reproductive endocrinology and infertility curriculum through three interactive cases. *MedEdPORTAL*. 2023;19:11375.
https://doi.org/10.15766/mep_2374-8265.11375

Introduction

Although reproductive endocrinology and infertility (REI) training is a required component of OB/GYN residency, the majority of clinical experience caring for patients with REI conditions may be limited to a single rotation lasting a few weeks in a 4-year residency.[1] The current number of REI subspecialists is inadequate to meet the increasing demand for infertility treatment in the United States, worsening wealth disparities and barriers to fertility care.[2,3] Although an estimated 17% of patients are impacted by infertility, less than a quarter receive treatment.[4] Despite limited training, general OB/GYNs serve as the frontline providers for clinic-based care for patients with infertility, and

Copyright © 2023 Kornfield et al. This is an open-access publication distributed under the terms of the Creative Commons Attribution license.

their skills are integral to meeting this need when subspecialists are not accessible or available. To maximize patient access to fertility care, it is imperative for general OB/GYNs to be proficient in the initial evaluation and management of fertility-related conditions, as laid out by the Council on Resident Education in Obstetrics and Gynecology (CREOG) learning objectives.[5] Moreover, as controlled ovarian hyperstimulation and oocyte retrieval for in vitro fertilization (IVF) and oocyte cryopreservation are increasing in frequency,[6] it is likely that general OB/GYNs will encounter postoperative complications as emergency department consultants. Therefore, it is critical that generalist OB/GYNs have proficiency to manage these scenarios.

Overall, there is a need for improved REI education among OB/GYN residents. A needs assessment conducted by our group highlighted this both nationally and specifically at our institution. Studies suggest that OB/GYN residents possess limited self-perceived and objectively assessed REI content knowledge.[1,7,8] In one study, poor polycystic ovary syndrome knowledge was only partially improved by completing an REI rotation, supporting a need for additional clinical training time and didactics throughout residency.[8] Residents surveyed at our institution reported low confidence in managing REI conditions and perceived REI as unusually challenging: 62% of residents disagreed with the statement "I can diagnose REI issues," and 88% agreed with the statement "REI is challenging."[9] Historically, national annual CREOG resident competency assessment exam scores have been low in REI subspecialty content.[10]

At our institution, in-service training exam scores on REI-related topics track with the national average, and postgraduation years 1 and 5 graduates scored their preparation in REI as fourteenth out of 15 topics. A high proportion of postgraduation respondents report discomfort with REI: 56% report feeling "not well at all" or "slightly well" prepared in REI in practice. The existing curriculum at our institution includes one 6-week intern rotation with assigned independent reading, two formal didactics sessions as part of annual in-service exam preparation, and the recent addition of a 1-hour interactive game show–style didactic.[9] We sought to further bolster our resident REI education through a case-based curriculum. Limited published educational interventions exist for REI content. One study assessing case-based curricula on REI topics has been published; however, the educational content itself is not available for review or utilization by residency programs.[1] The one curriculum on REI topics in *MedEdPORTAL* was published over a decade ago.[11] Given the significant changes in clinical management over that time period, we identified the need for a new case-based curriculum in REI to maintain medical applicability and accuracy. Thus, we developed a new case-based curriculum to meet this need.

Methods

Curriculum Development

We utilized the six-step approach as outlined by Kern and colleagues to develop a facilitator-led case-based REI curriculum at our institution for OB/GYN residents.[12] We began the approach with a needs assessment, as described above. We developed learning objectives and educational strategies. Our objectives targeted not only knowledge, as is typical with traditional didactics, but also comprehension, application, and analysis, in accordance with Bloom's taxonomy.[12] We chose case-based interactive learning for the curriculum because it is an established effective educational strategy, particularly for translating knowledge to clinical applicability.[12] As even our senior residents and recent graduates had reported limited knowledge/confidence in REI topics, we did not think a completely nondirective approach would be successful. We designed a curriculum that utilized facilitators to guide learners through a hybrid of didactic and learner-centered democratic teaching styles. Our residency had had limited success incorporating flipped classroom with prereading before didactics, as few learners completed the prereading, and so, we designed a curriculum that could be effective without it. Ultimately, we developed a three-scenario case-based curriculum for use in facilitated groups with in-person and virtual flexibility. While our target audience was OB/GYN residents, we also included medical students rotating on the REI elective during the study period.

The predominant focus of an REI curriculum, defined by the CREOG learning objectives, is the outpatient office setting.[5] Corresponding to these objectives, the topics we chose for this curriculum included two office practice cases, each representing a common cause of secondary amenorrhea with patients presenting for desired fertility. These cases addressed four of the seven topics for which CREOG recommended residents should be able to describe testing, treatment, and management. We also recognized a knowledge gap in the management of urgent complications related to REI procedures. In many hospitals, REI physicians are not immediately available for consultation. Therefore, our third case was an emergency room scenario to educate learners on oocyte retrieval complications. Our goal was that generalist OB/GYNs would develop proficiency to manage these complications, as expected for all other OB/GYN procedures. This represented a novel contribution to existing REI curricula.

Copyright © 2023 Kornfield et al. This is an open-access publication distributed under the terms of the Creative Commons Attribution license.

Initial Curriculum Pilot

The curriculum was deemed to be educational quality improvement by our institutional review board (IRB) and was exempt from requiring IRB approval. The three scenarios and pre- and postintervention assessments were reviewed by four content experts (two REI faculty and two REI fellows). We did a test run of the cases with two OB/GYN fellows from non-REI subspecialties, with an REI fellow as the facilitator. During this test run, one of the fellows was in person and the other was present virtually to ensure feasibility. We incorporated feedback to improve the curriculum.

Implementation of Curriculum

This educational intervention was intended to last 2 hours during OB/GYN resident didactics. We included all present OB/GYN residents in addition to the medical student on an REI elective in the intervention. We subsequently trialed the curriculum virtually as well, with a resident on the REI rotation and a medical student on an REI elective (both were not present during the in-person intervention). OB/GYN residents required no prerequisite knowledge, clinical rotations, or prereading. For medical students, prerequisite knowledge included prior completion of their core OB/GYN clerkship and current participation in an REI elective. For the in-person session, 16 residents were split into two groups of eight, each group with an REI fellow as facilitator. Learners received a stapled packet—the learner guide—which consisted of the preintervention assessment, the set of cases without answers included, and the postintervention assessment (Appendix A).

Learners were allotted 10 minutes to complete the preintervention assessment. Facilitators were provided with a key that included the cases with detailed responses. Within each group, residents took turns reading a question and offering their own answer and rationale. Questions were open-ended and included developing a differential, interpreting diagnostic test results, and outlining appropriate treatments. If a learner was unsure of a response, facilitators encouraged other learners to offer input through group discussion. The facilitators ensured that residents answered questions accurately and provided them with guidance to complete responses (i.e., by considering all potential diagnoses). All information required by the facilitators to assist the residents was contained in the facilitator guide (Appendix B). Following completion of the cases, learners were allotted 10 minutes to complete the postintervention assessment. The pre- and postintervention assessments were removed from the packet and handed to the facilitator at conclusion of the session. The facilitator guide was subsequently distributed to the residents as a resource for future reference.

For the virtual group, the cases were completed as above, except that the learners and facilitator received electronic copies of the learner guide via email (Appendix A). Learners filled in their pre- and postintervention assessments using their preferred computer editing software. We used our institution's videoconferencing software, WebEx, although the curriculum could be conducted on any videoconferencing software offering screen sharing. The facilitator shared the learner guide (Appendix A) on the screen and privately also had the facilitator guide (Appendix B), which they used to guide the discussion, open. Following the session, learners emailed their entire completed learner guide (Appendix A), including assessment responses, to the facilitator.

Curriculum Evaluation

We used written assessments to obtain quantitative and qualitative information on cognitive and affective outcomes. Participants completed three assessments: directly before the intervention, immediately at the completion of the intervention, and 7 weeks following the in-person session (3 weeks following the virtual session). Initial pre- and postintervention assessments were included at the front and back of the learner guide, respectively (Appendix A). The delayed postintervention assessment was solicited via a follow-up email sent simultaneously to all learners who had participated (Appendix C).

We assessed knowledge via three multiple-choice questions, each highlighting a learning point from one of the cases. The questions were written and evaluated by the four content experts and further edited following a test run with non-REI fellows. Identical question texts were used at all time points. The remainder of the questions were self-assessment of affective (confidence) and psychomotor (competence) outcomes. Questions utilized a Likert scale with 1 as the most negative response, 3 as neutral, and 5 as the most positive response. Learners were asked how comfortable they felt with the evaluation and management of three different possible patient presentations addressed in the curriculum. To identify whether low self-perceived knowledge/competence was universal to OB/GYN subspecialties or unique to REI, learners were also asked how both their knowledge and competence in REI compared to other subspecialties. On the immediate postassessment only, additional questions assessed satisfaction with the curriculum. Qualitative questions allowed for short answers, such as whether this intervention would change their practice. The delayed (7- or 3-week) posttest consisted of only the three knowledge questions and the assessment of self-perceived knowledge and competence in REI compared to other subspecialities. This delayed assessment was intentionally

Copyright © 2023 Kornfield et al. This is an open-access publication distributed under the terms of the Creative Commons Attribution license.

abbreviated to maximize potential responses, as a low response rate was anticipated via email based on prior similar interventions in our residency. Immediate pre- and postassessments were anonymous but linked together; however, the delayed assessment was neither anonymous nor linked by participant to the previous responses.

Statistical Analysis
Pre- and immediate postassessment responses were compared with paired *t* tests or McNemar tests, as appropriate.

Results

Eighteen learners participated in the intervention. The in-person cohort contained 16 learners: 15 OB/GYN residents and one medical student. The virtual cohort contained two learners: one medical student and one first-year resident. The distribution among learning levels for all participants in the intervention is displayed in Table 1. One learner completed the preintervention assessment but did not complete the immediate postintervention assessment at all. Four additional learners variably completed the postintervention assessment. Therefore, 17 of the 18 participants (94%) responded to at least some of the questions, and 13 (72%) responded to all questions. A learner's responses were included in the statistical analysis only if the learner had both a pre- and postintervention assessment response. Therefore, the number of respondents ranged from 13 to 17 out of 18 by question. Eight participants completed the delayed postintervention assessment.

Preintervention and immediate postintervention assessment results are presented in Table 2. From preintervention to immediate postintervention assessments, learners demonstrated significant improvements in their self-assessed comfort with both the evaluation and management of three REI conditions: irregular menses, infertility, and secondary amenorrhea ($p \leq .01$ for all). Learners on average reported their REI knowledge and comfort as worse than other OB/GYN subspecialties on the preintervention assessment, with mean scores of 2.3 and 2.2, respectively. Postintervention, self-perceived REI knowledge and comfort improved significantly, to 3.4 and 3.1, respectively ($p < .001$ for both). Respondents had improved performance on all knowledge-based questions postintervention, and this was statistically significant for two of the three questions ($p < .05$ for questions 1 and 2, $p = .16$ for question 3).

Table 1. Participants' Education Levels

Training Year	No. (%)
Medical student	2 (11)
First-year resident	5 (28)
Second-year resident	4 (22)
Third-year resident	3 (17)
Fourth-year resident	4 (22)
Total	18 (100)

We conducted a sensitivity analysis in which the two virtual learners were excluded from the analysis. This did not alter the results, suggesting that inclusion of the virtual learners did not skew our findings. Virtual participant numbers were insufficient to perform comparative statistics with in-person learners.

On the immediate postintervention assessment, 100% of respondents (16 of 16) reported satisfaction with and enjoyment of the curriculum. Satisfaction was assessed on a 5-point scale (1 = *not satisfied at all,* 5 = *extremely satisfied*). Of respondents, 12% (two of 16) chose 4 (*very satisfied*), and 88% (14 of 16) chose 5 (*extremely satisfied*). Enjoyment was also assessed on a 5-point scale (1 = *did not enjoy at all,* 5 = *enjoyed very much*). Of respondents, 12% (two of 16) chose 4 (*mostly enjoyed*), and 88% (14 of 16) chose 5 (*enjoyed very much*).

Responses to qualitative questions were predominantly positive. Trends included learners reporting that the cases were clinically relevant and applicable to their practice and that the format was engaging. One learner requested more specific diagrams on related topics, plus a sheet of take-home points. A medical student reported that the cases would have been too difficult if she had not been on her REI rotation.

Eight respondents participated in the delayed postintervention assessment. Respondents consisted of one medical student and seven residents: three first-years, two second-years, one third-year, and one fourth-year. Responses demonstrated sustained improvement in self-perceived knowledge and comfort, as well as improved scores on the knowledge-based questions (Table 3). However, low response rate and lack of paired statistical analysis limited this assessment.

Discussion

This publication describes the implementation of a case-based curriculum that effectively improved learners' REI objective knowledge and subjective confidence at our institution. Qualitative feedback was overwhelmingly positive, and improvements in knowledge persisted even following a 7-week delay. Additionally, the utilization of a small-group learning environment supported by a facilitator enabled this curriculum to be implemented without the need for senior resident competency in this content area.

There are only a few previously published REI curricula available, and only one within the last 5 years.[1,9,11] Importantly, our curriculum is up to date on current practice guidelines in a

Copyright © 2023 Kornfield et al. This is an open-access publication distributed under the terms of the Creative Commons Attribution license.

Table 2. Learner Responses on Pre- and Postintervention Assessments

Item	N	Preintervention		Postintervention		p[a]
		M (SD)	No. (%)	M (SD)	No. (%)	
How comfortable do you feel *evaluating* a patient with[b]						
Irregular menses	16	3.9 (0.9)		4.4 (0.5)		.01
Infertility	16	3.3 (0.6)		4.2 (0.5)		<.001
Secondary amenorrhea	17	3.2 (0.6)		3.9 (0.7)		<.001
How comfortable do you feel *managing* a patient with[b]						
Irregular menses	16	3.6 (0.9)		4.1 (0.5)		.003
Infertility	16	2.6 (0.6)		3.8 (0.4)		<.001
Secondary amenorrhea	16	2.8 (0.6)		3.8 (0.5)		<.001
How do you feel your reproductive endocrinology and infertility *knowledge* and *comfort* compare to other subspecialities[c]						
Knowledge	16	2.3 (0.7)		3.4 (0.6)		<.001
Comfort	16	2.2 (0.7)		3.1 (0.7)		<.001
Percentage of correct responses to multiple-choice knowledge questions						
Knowledge question 1	13		9 (69)		13 (100)	.04
Knowledge question 2	13		7 (54)		12 (92)	.02
Knowledge question 3	13		11 (85)		13 (100)	.16

[a]Paired *t* test/McNemar test used.
[b]Rated on a 5-point Likert scale (1 = *not comfortable at all*, 5 = *very comfortable*).
[c]Rated on a 5-point Likert scale (1 = *significantly worse*, 5 = *significantly better*).

rapidly changing field, and two of its three cases focus on essential CREOG learning objectives. Additionally, the third case includes an REI-themed emergency department consultation, whereas most emphasis in the REI curricula relates only to office practice. Although complications after oocyte retrievals are not frequently encountered, their management can fall on generalist OB/GYNs, and IVF/oocyte retrieval complications have unique characteristics that differentiate them from other surgical complications. While this topic is not currently included in the CREOG learning objectives, our curriculum offers a unique and novel opportunity to review this material with OB/GYN residents.

Although we primarily studied this curriculum in an in-person learning environment, we did verify that it was possible to implement it both in person and virtually. While virtual learning is often utilized out of necessity, in-person learning likely promotes better engagement.[13] As we have insufficient data to directly compare virtual to in person, we would recommend in-person use of the curriculum if possible, with virtual implementation as an acceptable alternative. It is important that REI curricula include a virtual option, as many OB/GYN programs do not have REI faculty at their primary hospital site and in-person requirements can limit access to these subspecialists. If implemented virtually, the documents can be screen shared over any videoconference software, as trialed in our test run and piloted with one facilitator and two learners for each. Due to potential for decreased engagement in a virtual format, smaller groups would likely be preferred—probably up to four learners to a facilitator—to encourage participation and engagement.

Table 3. Learner Responses on Delayed Postintervention Assessment

Item	Delayed Postintervention	
	M (SD)	No. (%)
How do you feel your reproductive endocrinology and infertility *knowledge* and *comfort* compare to other subspecialities[a]		
Knowledge	3.3 (1.0)	
Comfort	3.0 (0.8)	
Percentage of correct responses to multiple-choice knowledge questions[b]		
Knowledge question 1		6 (75)
Knowledge question 2		7 (88)
Knowledge question 3		7 (88)

[a]Rated on a 5-point Likert scale (1 = *significantly worse*, 5 = *significantly better*).
[b]$N = 8$.

A potential limitation of our curriculum is its use of facilitators, a more resource-intensive educational approach. We used REI fellows, who often had more availability than faculty. We also successfully implemented the curriculum in person in larger groups of eight, which allowed for fewer facilitators. For residencies without REI fellows, it would be reasonable to substitute either REI faculty or a general OB/GYN with REI interest. Practicing general OB/GYNs at our institution reviewed the facilitator guide and felt they would be sufficiently prepared to teach the course. Additionally, given the provided facilitator guides, anticipated time commitment is only 2 hours, with minimal preparation needed.

We encountered barriers in our administration of curriculum assessments. Some respondents did not complete the immediate postintervention assessment in its entirety, potentially due to errors noted in preparation of some of their learner guides, which omitted and misordered questions. In addition, response rates were lower on the delayed postintervention assessment despite its brevity. Because pre- and postintervention assessments

Copyright © 2023 Kornfield et al. This is an open-access publication distributed under the terms of the Creative Commons Attribution license.

were linked on paper but anonymous, we were unable to perform paired analyses to compare individuals' responses across survey time points. Missing postintervention responses could have biased our results if those learners had a negative experience and thus were less likely to complete the delayed assessment. Additional limitations were a smaller sample size and a brief knowledge assessment of three questions. In addition, improvement on the third question did not reach statistical significance, likely due to sample size and a high proportion of learners (85%) responding correctly on the pretest. We intentionally kept the knowledge quiz short to maximize learner responses but acknowledge this tested only a small fraction of the material covered in the curriculum.

This curriculum represents a valuable contribution to the field, given that no clinically accurate case-based learning curricula are currently available in *MedEdPORTAL*. Although we have implemented this curriculum with only a small number of learners in a single institution, it could be easily incorporated by other OB/GYN residencies, even without access to in-person REI faculty. While it would be too advanced for a general medical school curriculum, it is also appropriate for students on an REI elective. We lack data highlighting improved clinical practice or patient care, but learners reported that the curriculum was clinically relevant and translatable. In our institution, we intend to utilize these cases every other year, such that OB/GYN residents are exposed twice over the 4-year residency. We hope that with continued growth in our REI didactics curriculum in addition to this intervention, we will see improvements not only in annual CREOG scores but also on postgraduation surveys. Our larger goal is that through improving OB/GYN residency training, we can increase generalist OB/GYN competency and confidence in this field to improve patient REI care and access.

Appendices

A. Learner Guide and Pre- and Postsurveys.docx

B. Facilitator Guide.docx

C. Delayed Postsurvey.docx

All appendices are peer reviewed as integral parts of the Original Publication.

Molly Siegel Kornfield, MD: Fellow Physician, Division of Reproductive Endocrinology and Infertility, Department of Obstetrics and Gynecology, Oregon Health & Science University School of Medicine; ORCID: https://orcid.org/0000-0001-5005-1840

Elizabeth Rubin, MD: Assistant Professor, Division of Reproductive Endocrinology and Infertility, Department of Obstetrics and Gynecology, Oregon Health & Science University School of Medicine; ORCID: https://orcid.org/0000-0002-7567-0151

Pamela Parker, MD, MPH: Assistant Professor, Department of Obstetrics, Gynecology, and Reproductive Sciences, University of Pittsburgh School of Medicine; ORCID: https://orcid.org/0000-0003-2412-3614

Bharti Garg, MBBS, MPH: Biostatistician, Department of Obstetrics and Gynecology, Oregon Health & Science University School of Medicine; ORCID: https://orcid.org/0000-0002-6731-0623

Amy Stenson, MD, MPH: Associate Professor, Department of Obstetrics and Gynecology, Oregon Health & Science University School of Medicine

Paula Amato, MD: Professor, Division of Reproductive Endocrinology and Infertility, Department of Obstetrics and Gynecology, Oregon Health & Science University School of Medicine

Sacha Krieg, MD, PhD: Associate Professor, Division of Reproductive Endocrinology and Infertility, Department of Obstetrics and Gynecology, Oregon Health & Science University School of Medicine

Acknowledgments
The authors thank Katherine Jorda and Fatima Salas for their assistance in obtaining resident postgraduation survey data.

Disclosures
None to report.

Funding/Support
None to report.

Ethical Approval
The Oregon Health & Science University Institutional Review Board deemed further review of this project not necessary.

References

1. Goldman KN, Tiegs AW, Uquillas K, et al. Interactive case-based learning improves resident knowledge and confidence in reproductive endocrinology and infertility. *Gynecol Endocrinol*. 2017;33(6):496-499. https://doi.org/10.1080/09513590.2017.1290075
2. Komorowski AS, Jain T. A review of disparities in access to infertility care and treatment outcomes among Hispanic women. *Reprod Biol Endocrinol*. 2022;20:1. https://doi.org/10.1186/s12958-021-00875-1
3. Brodeur TY, Grow D, Esfandiari N. Access to fertility care in geographically underserved populations, a second look. *Reprod Sci*. 2022;29(7):1983-1987. https://doi.org/10.1007/s43032-022-00991-2
4. Boivin J, Bunting L, Collins JA, Nygren KG. International estimates of infertility prevalence and treatment-seeking: potential need

Copyright © 2023 Kornfield et al. This is an open-access publication distributed under the terms of the Creative Commons Attribution license.

and demand for infertility medical care. *Hum Reprod*. 2007;22(6): 1506-1512. https://doi.org/10.1093/humrep/dem046

5. Council on Resident Education in Obstetrics and Gynecology. *Educational Objectives: Core Curriculum in Obstetrics and Gynecology*. 13th ed. American College of Obstetricians and Gynecologists; 2022. Accessed November 7, 2023. https://www.acog.org/-/media/project/acog/acogorg/files/creog/creog-educational-objectives-13th-edition.pdf
6. Assisted reproductive technology (ART): ART success rates. Centers for Disease Control and Prevention. Updated September 25, 2023. Accessed November 7, 2023. https://www.cdc.gov/art/artdata/index.html#preliminary
7. Roberts LM, Kudesia R, Zhao H, Dolan S, Rose M. A cross-sectional survey of fertility knowledge in obstetrics and gynecology residents. *Fertil Res Pract*. 2020;6:22. https://doi.org/10.1186/s40738-020-00091-2
8. Jackson-Bey T, Mehr H, Ho JR, et al. Provision of infertility care for the underserved in reproductive endocrinology and infertility practices associated with obstetrics and gynecology residency training programs in the United States. *F S Rep*. 2022;3(2)(suppl): 106-113. https://doi.org/10.1016/j.xfre.2021.11.002
9. Parker PB, Krieg SA. A virtual, interactive quiz-show curriculum is associated with increased resident knowledge in reproductive endocrinology and infertility. *Am J Obstet Gynecol Res*. 2022;2(1):1007. https://www.medtextpublications.com/open-access/a-virtual-interactive-quiz-show-curriculum-is-associated-with-increased-resident-1102.pdf
10. Holzman GB, Downing SM, Power ML, Williams SB, Carpentieri A, Schulkin J. Resident performance on the Council on Resident Education in Obstetrics and Gynecology (CREOG) In-Training Examination: years 1996 through 2002. *Am J Obstet Gynecol*. 2004;191(1):359-363. https://doi.org/10.1016/j.ajog.2004.01.028
11. Houmard B, McLemore L, Chiang S. Resident-guided case discussions in reproductive endocrinology & infertility. *MedEdPORTAL*. 2012;8:8494. https://doi.org/10.15766/mep_2374-8265.8494
12. Thomas PA, Kern DE, Hughes MT, Chen BY, eds. *Curriculum Development for Medical Education: A Six-Step Approach*. 3rd ed. Johns Hopkins University Press; 2016.
13. Fedoruk K, Abir G, Carvalho B. Virtual compared to in-person obstetric anesthesiology trainee education during the COVID-19 pandemic: a short report. *Cureus*. 2022;14(6):e26423. https://doi.org/10.7759/cureus.26423

Received: March 23, 2023
Accepted: October 23, 2023
Published: December 21, 2023

Copyright © 2023 Kornfield et al. This is an open-access publication distributed under the terms of the Creative Commons Attribution license.
